# 

**Published:** 1986-08

**Authors:** 


VOLUME 101(iv) AUGUST 1986

NATIONAL LIBRARY
JUN 22 1987
OF MEDICINE

No fault compensation for accidental injury. p 77
Diabetes Mellitus in Mental Handicap. p 82
Upper G–I Endoscopy. p 83
The Green paper. An hypothesis to explain the delay in its appearance. p 88
News and Comment. p 85
And Much Else

IN ASSOCIATION WITH

### BRISTOL MEDICINE

THE MEDICAL JOURNAL OF THE SOUTH WEST

